# Influenza like Illness among Medical Residents Anticipates Influenza Diffusion in General Population: Data from a National Survey among Italian Medical Residents

**DOI:** 10.1371/journal.pone.0168546

**Published:** 2016-12-20

**Authors:** Vincenzo Restivo, Claudio Costantino, Caterina Mammina, Francesco Vitale

**Affiliations:** Department of Sciences for Health Promotion and Mother-Child Care “G. D’Alessandro”, University of Palermo, Palermo, Italy; The Scripps Research Institute, UNITED STATES

## Abstract

The aim of this multicentre study was to assess incidence of influenza like illness (ILI) among Italian medical residents (MRs) during 2011–2012 influenza season, to detect variables associated with ILI and to compare estimated ILI incidence among MRs and general population. A cross-sectional survey was carried out throughout an anonymous questionnaire administered to all MRs attending the post-graduate medical schools of 18 Italian Universities. At the same time an analysis of the ILI incidence in the Italian general population was conducted through the Italian Influenza Surveillance Network. Of a total of 2,506 MRs, 1,191 (47.5%) reported at least one ILI episode. A higher proportion of ILIs was reported by MRs of Central (25.0% with ILI vs 20.2% without ILI) and Southern Italy (40.2% with ILI vs. 36.4 without ILI) compared to Northern Italy (34.8% with ILI vs. 43.4% without ILI) (p<0.001). Italian MRs had a higher cumulative incidence of ILIs (546.7 episodes per 1,000 vs. 75.9 episodes per 1,000) and an earlier peak (January 2012 vs. February 2012), compared to general population due to higher number of contacts in hospital setting. MRs reported a high rate of ILI infection probably in association with their working activities. These data suggest the need to offer an earlier influenza vaccination to HCWs than general population with the aim to both prevent ILI and its transmission to patients.

## Introduction

The assumption of influenza transmission from patients to healthcare workers (HCWs) and vice-versa seems obvious, since influenza is primarily transmitted by infected persons, and HCWs have increased risk of daily close contact during the influenza season [1]. Despite concern for hospital-acquired infection and its impact on vulnerable populations, limited data are available on influenza like illness (ILI) incidence during influenza season among a special category of HCWs, i.e. medical residents (MRs) [2]. MRs, indeed, can be frequently the first contact for patients, but they can lack knowledge and training about adoption of preventive measures [3–5]. Moreover, MRs who are in charge of the hospital patient care may play a key role in fostering appropriate preventive behaviors through the patients [3].

Several studies have reported variable attack rates during influenza epidemics, fluctuating from 0.7% to 20% among patients and from 11% to 59% among HCWs [1,6]. The risk assessment of acquiring influenza in HCWs seems complex because of difficulties in monitoring HCWs during their daily activities [7]. It is proved that infected HCWs continue to work fostering influenza transmission, even to debilitated patients [8]. Moreover, two studies analyzing index cases in an infectious diseases and in a solid organ transplant units, suggest that HCWs are more likely to acquire influenza than hospitalized patients [7,9]. Lastly, the increase of hospital admissions during influenza season is typically concurrent with occupational infections [1]. Coley et al. have supposed that the secondary attack rate among unprotected HCWs is higher (54.3%) respect to attack rate among adults (34.1%) [10]. Nonetheless, only few studies have been carried out about ILI incidence in MRs at both the local and national level [3]. The objective of this multicentre study were to assess the reported incidence of ILIs among Italian MRs during the 2011–2012 influenza season and to detect variables associated with ILI occurrence. Additionally, the estimated ILI incidences among MRs and general population were compared.

## Methods

This cross-sectional study was carried out between April and June 2012 and involved all MRs attending the post-graduate medical schools of 18 Italian Universities (Bari, Bologna, Brescia, Catania, Catanzaro, Chieti, L’Aquila, Messina, Modena, Napoli Federico II, Palermo, Pavia, Parma, Roma Cattolica, Roma Tor Vergata, Siena, Torino, Verona). A total of 10,854 were asked for participating to the study, after obtaining their e-mail addresses from the respective University administrative offices. MRs without at least one working e-mail address were ruled out from the study. Each MR enrolled was recruited by sending her/him an e-mail containing a description of the objectives and methods of the study, an informed consent form and a link to a self administered, anonymous questionnaire. After 30 days, a reminder e-mail was sent to non-responders. The study was approved by the Ethical Committee of the Azienda Ospedaliero-Universitaria Policlinico “P. Giaccone”, Palermo, Italy.

The questionnaire had been previously designed by a national working group, piloted during a previous study and used in a local survey [4]. The final version of the questionnaire included 10 sections with 23 items. Data concerning reasons of influenza vaccination uptake or refusing, as well as attitudes to recommend influenza vaccination to patients, had been analyzed elsewhere [11]. In the present manuscript, in particular, four sections of the questionnaire were considered, which were related to ILI incidence and prevention:

1. socio-demographic and academic characteristics: gender, age, year of graduation, year of residency, specialty duties (categorized according to Italian law as “clinical”, “surgical” and “diagnostic”), geographic setting (categorized as “Northern”, “Central” and “Southern” Italy);
2. number of ILI episodes during the 2011–2012 influenza season (from November 1^st^ 2011 to March 31^th^ 2012) and month of onset (comprehensive instructions about ILI definition according to European Centre for Diseases Prevention and Control were provided in line with the web-based questionnaire) [12];
3. influenza vaccination coverage during the 2009–2010 (categorized as “pandemic AH1N1”, “seasonal”, “both pandemic AH1N1 and seasonal”), 2010–2011 and 2011–2012 influenza seasons;
4. personal attitudes relating to respiratory disease transmission: washing hands’ frequency (categorized as “a maximum of 3”, “from 4 to 6” and “at least 7” times in a day), regular use of a personal protective equipment and smoke habits.

ILI incidence in the general Italian population aged 15–64 years was obtained from the Italian Influenza Surveillance Network (InfluNet), coordinated by the National Health Institute (ISS), Rome, Italy [13]. The target of such a surveillance network is a representative sample of at least 2% of the general Italian population enrolled through a sample of general practitioners (GPs) scattered all over the country. Data from the 11 regions where the questionnaire was administered were extracted for a total of 1,196,109 people under surveillance. According to the InfluNet definitions, an influenza season was defined as the period included between the 42^nd^ week of each year and the 17^th^ week of the following year [14]. Indeed, data from the ECDC surveillance system have confirmed that this interval of time accurately defines the period between the first and last case of influenza in the community. [12]

### Statistical Analysis

All data were analyzed by using STATA 11.3 statistical software package. Absolute and relative frequencies were calculated for qualitative variables, while quantitative variables were summarized as median (interquartile range, IQR). Categorical variables were analyzed using the chi-square test while the continuous using the Mann-Whitney-Wilcoxon test. The significance level was set at p < 0.05.

Data on ILI spread in the general Italian population and in MRs were presented as cumulative incidence (cases/month). Trend in annual influenza vaccination coverage was evaluated by Chi-square for trend. To evaluate difference among incidences was used a tests of hypothesis based on the Poisson distribution with normality approximation.

## Results

A total of 2,506 out of 10,854 MRs were recruited in the survey. The response rate was 24.1% ranging between a minimum of 20.3% in Parma and a maximum of 28.7% in Bologna. The respondents had a median age of 29 years (IQR 28–31). The majority were of female gender (64.7%), had clinical duties (43.8%) and was attending the first three years of residency (84.3%). The geographic distribution showed a larger number of respondents in the Northern and Southern medical schools (39.3% and 38.2%, respectively) compared to Central Italy (22.5%) (Table 1).

**Table 1:** Socio-demographic and academic characteristics of the Italian medical residents (MRs) recruited in this study.

Characteristic	Total MRs 2,506
**Gender, n (%)**	
- male	885 (35.3)
**Age class in year, n (%)**	
- ≤ 29	1,300 (51.9)
- > 29	1,206 (48.1)
**Specialty duties, n (%)**	
- Clinical	1,099 (43.8)
- Surgical	567 (22.6)
- Diagnostic	840 (33.6)
**Geographical distribution, n (%)**	
- Northern Italy	985 (39.3)
- Central Italy	564 (22.5)
- Southern Italy	957 (38.2)

*IQR, interquartile range

About the preventive behaviors against influenza, 472 MRs (18.8%) declared they were not using protective equipment during their healthcare activities and 551 MRs (22.0%) having smoked at least 100 cigarettes during their lifetime. In 1,698 cases (67.8%), MRs reported washing their hands more than seven times daily. Five hundred forty-three (21.7%) participants declared to have got influenza vaccine in the 2009–2010 season (at least one among “pandemic AH1N1”, “seasonal”, “both pandemic AH1N1 and seasonal”), with a steadily decrease to 15.5% in the 2010–2011 and 11.9% in 2011–2012 seasons (Table 2).

**Table 2:** Preventive attitudes and behaviors against influenza among the Italian medical residents (MRs) recruited in this study.

	Total MRs 2,506
**Hand washing frequency, n (%)**	
- less than 3 times/day	116 (4.6)
- from 4 to 6 times/day	692 (27.6)
- more than 7 times/day	1,698 (67.8)
**Personal protective equipment use, n (%)**	
- habitually	1,390 (55.5)
- always	644 (25.7)
- never	472 (18.8)
**Influenza vaccination 2009/2010, n (%)**	
- Yes	543 (21.7)
- No	1,963 (78.3)
**Influenza vaccination 2010/2011, n (%)**	
- Yes	388 (15.5)
- No	2,118 (84.5)
**Influenza vaccination 2011/2012, n (%)**	
- Yes	299 (11.9)
- No	2,207 (88.1)
**Smoking habits, n (%)**	
- Yes, more than 100 cigarettes/lifetime	551 (22.0)
- No	1,955 (78.0)

Among the surveyed MRs, 1,190 (47.5%) reported at least one ILI episode during the 2011–2012 influenza season. In Table 3 socio-demographic and academic characteristics, preventive attitudes and behaviors against influenza of these MRs were compared with those of MRs reporting no ILI episodes.

**Table 3:** Characteristics of the Italian medical residents (MRs) that reported or not at least one episode of Influenza-like Illness (ILI) during 2011/2012 influenza season.

	with ILI*1,190	without ILI*1,316	p-value
**Gender, n (%)**			
- male	402 (33.8)	483 (36.7)	0.13
- female	788 (66.2)	833 (63.3)	
**Age class in year, n (%)**			
- ≤ 29	632 (53.1)	668 (50.8)	0.24
- > 29	558 (46.9)	648 (49.2)	
**Specialty duties, n (%)**			
- Clinical	541 (45.5)	558 (43.4)	0.25
- Surgical	256 (21.5)	311 (23.6)	
- Diagnostic	393 (33.0)	447 (33.0)	
**Geographical distribution, n (%)**			
- Northern Italy	414 (34.8)	571 (43.4)	<0.001
- Centre Italy	298 (25.0)	266 (20.2)	
- Southern Italy	478 (40.2)	479 (36.4)	
**Smoking habits, n (%)**			
- yes, more than 100 cigarettes/lifetime	265 (22.3)	286 (21.7)	0.75
- no	925 (77.7)	1,030 (78.3)	
**Hand washing frequency, n (%)**			
- less than 3 times/day	56 (4.7)	60 (4.6)	0.64
- from 4 to 6 times/day	318 (26.7)	374 (28.4)	
- more than 7 times/day	816 (68.6)	882 (67.0)	
**Personal protective equipment use, n (%)**			
- habitually	660 (55.5)	730 (55.5)	0.90
- always	302 (25.4)	342 (26.0)	
- never	228 (19.1)	244 (18.5)	
**Influenza Vaccination 2009/2010, n (%)**			
- Yes	260 (21.8)	283 (21.5)	0.83
- No	930 (78.2)	1,033 (78.5)	
**Influenza Vaccination 2010/2011, n (%)**			
- Yes	191 (16.0)	197 (15.0)	0.45
- No	999 (84.0)	1,119 (85.0)	
**Influenza Vaccination 2011/2012, n (%)**			
- Yes	139 (11.7)	160 (12.2)	0.71
- No	1,051 (88.3)	1,156 (87.8)	

*ILI, Influenza-like Illness.

ILI episodes occurred more frequently among female MRs (female gender 66.2% vs. male gender 33.8%, p = 0.13) and among MRs with clinical duties (clinical 45.5% vs. other 54.5%, p = 0.25). A higher proportion of ILIs was reported by MRs of Central (25.0% with ILI vs 20.2% without ILI) and Southern Italy (40.2% with ILI vs. 36.4 without ILI) compared to Northern Italy (34.8% with ILI vs. 43.4% without ILI) (p<0.001). No statistically significant differences were detected between MRs reporting ILI and those who did not about their smoking habits, daily hand washing frequency, personal protective equipment use and influenza vaccine uptake in the seasons under consideration.

**Fig 1:**
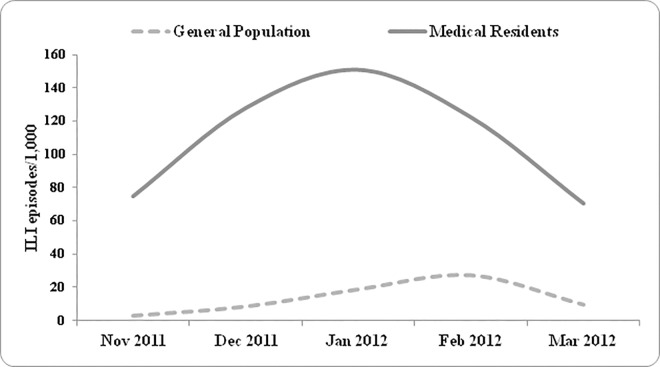
Influenza like-illness incidence during the 2011/2012 influenza season among general population and the Italian medical residents.

Fig 1 describes the cumulative incidence (chi-square for trend 2.61, p <0.01) of the reported ILIs among Italian MRs (546.7 episodes per 1,000), in comparison with the general population incidence (75.9 episodes per 1,000). ILIs within MRs shows a rising trend since November 2011 with 74 episodes per 1,000, reaching a peak in January 2012 with 151.2 episodes per 1,000. Conversely, cumulative ILI incidence in the general population was low until December 2011 peaking in February 2012, with 27 episodes per 1,000 patients.

## Discussion

Since MRs can be the first-line care providers for hospitalized patients and, consequently, carry a high risk of getting from or transmitting influenza to patients, they should deserve a special consideration as an important HCWs group for investigating ILI incidence.

According with our study results, a higher interest towards influenza was showed by MRs attending the first three years of residency, a group at higher risk of acquiring ILI due to a great number of extra-work contacts with their peer and a lower immunity during younger age [15]. Except for influenza vaccination, MRs reported a widely adoption of protective behaviors. As well as other studies confirm, a large proportion of MRs reported low influenza vaccination coverage because of concerns about side effects, lack of perceived risk and inadequate university training [3,5,11].

In this national multicenter study, the reported incidence of ILIs among MRs was 47.5%. This rate is significantly higher than incidence reported in some European countries among HCWs, such as France (4.3%) and Germany (11.4%), and in other non-European countries like US (5.4%) and Canada (24.0%) [16–19]. This higher incidence among the Italian MRs was not linked to personal protective equipment use because Italian MR declared a positive attitude toward its adoption during clinical practice not dissimilarly to European and extra-European HCW. [19, 20] On the other hand the higher ILIs incidence could be related to lower vaccination rates observed in Italy than other countries (49.5% in US and 45.6% in France) [11,21,22]. Furthermore, a large majority of ILI cases was generally observed among under 25 year-old subjects, an age group which some MRs attending the first three years belong to [23].

The reported ILI incidence among Italian MRs was higher in Southern and Central than Northern Italy. On the other hand, ILI incidence among general population was higher in the Northern regions [13]. This observation is not skewed by a dissimilar distribution of the respondent MRs through the different macro-areas. Moreover, it is not related to a different vaccine coverage in these three geographic settings. These inconsistencies might suggest a different pattern of the influenza virus circulation, but they could be also attributed to a general misclassification of influenza and other respiratory virus diagnosis. At the least, our findings suggest a poor medical training on the epidemiology and diagnosis of ILI cases [24].

Interestingly, the reported ILI incidence peak among MRs was significantly earlier compared to general population. The increased risk of influenza and ILI among HCWs compared to general population is widely recognized and related to occupational exposure risk [3,25]. MRs, in particular, are a group of HCWs with frequent and close contacts with patients, aside from nurses and the ancillary staff, and frequently in charge of caring multiple patients even on different wards. Our results could also support findings of previous studies about possible ILI transmission before symptoms onset [24]. A further study shows that HCWs have a similar risk of acquiring ILI to the subjects sharing their households with children, that substantially increases the risk of infection [6]. Indeed it is well known from family-based studies that ILI incidence is higher in children than in adults, moreover observational studies have shown that living with children increases the risk for laboratory-confirmed influenza infection [6].

Finally, MRs show not only an earlier but also a disproportionately higher cumulative ILI incidence compared to general population (546.7 per 1,000 vs. 75.9 per 1,000). This supports a higher risk acquiring ILI among MRs even during non-epidemic influenza periods, suggesting their key role in transmitting ILI etiological agents to their patients and relatives [23]. Furthermore, overcrowding during the winter season promotes spreading of influenza viruses in healthcare settings [26]. Consequently, MRs engaged in wards such as internal medicine, emergency medicine, pediatrics, and obstetrics and gynecology, should be considered with special attention [3]. Furthermore, the low vaccination coverage and the high incidence of ILI in MRs, a peculiar subgroup of HCWs with a great number of close contact with general population, suggests to investigate GPs. Indeed GPs have larger contacts with the general population and administer influenza vaccine to high numbers of eligible subjects.

This study has two main limitation. First, ILI diagnosis in MRs was self-reported. Misclassification of ILI was minimized providing inside the self-administered questionnaire a complete and appropriate ILI definition, according with the ECDC guidelines. Second, an underestimation of ILI cases in the community was possible as a consequence of the active design of the Italian ILI surveillance system, where cases among general population need to consult a GP before to be diagnosed and notified. Consequently, comparison of ILI incidence among MRs and general population has to be interpreted cautiously.

Despite these limitations, this is the first national study examining ILI incidence among MRs attending postgraduate medical school in one of the most populated European countries. The findings of our work suggest the need to offer an earlier influenza vaccination to HCWs than general population with the purpose to both prevent ILI and its transmission to their patients. Although MRs reported a high rate of ILI infection likely due to their workplace exposure, many of them did not apparently comply with the universally recommended infection control measures. Moreover, ILI preventive behavioral strategies and influenza vaccination need to be more carefully considered during the degree and postgraduate medical courses.

## Supporting Information

S1 Dataset ILI among medical residents dataset. (XLS)
